# CCIVR2 facilitates comprehensive identification of both overlapping and non-overlapping antisense transcripts within specified regions

**DOI:** 10.1038/s41598-023-42044-x

**Published:** 2023-09-08

**Authors:** Maya Suzuki, Satoshi Sakai, Kosuke Ota, Yuki Bando, Chiharu Uchida, Hiroyuki Niida, Masatoshi Kitagawa, Tatsuya Ohhata

**Affiliations:** 1 Department of Molecular Biology, Hamamatsu University School of Medicine, Hamamatsu, Shizuoka 431-3192 Japan; 2 Department of Organ and Tissue Anatomy, Hamamatsu University School of Medicine, Hamamatsu, Shizuoka 431-3192 Japan; 3 Advanced Research Facilities and Services, Preeminent Medical Photonics Education and Research Center, Hamamatsu University School of Medicine, Hamamatsu, Shizuoka 431-3192 Japan

**Keywords:** Cell biology, Genetics

## Abstract

Pairs of sense and antisense transcriptions that are adjacent at their 5′ and 3′ regions are called divergent and convergent transcription, respectively. However, the structural properties of divergent/convergent transcription in different species or RNA biotypes are poorly characterized. Here, we developed CCIVR2, a program that facilitates identification of both overlapping and non-overlapping antisense transcripts produced from divergent/convergent transcription whose transcription start sites (TSS) or transcript end sites (TES) are located within a specified region. We used CCIVR2 to analyze antisense transcripts starting around the sense TSS (from divergent transcription) or ending around the sense TES (from convergent transcription) in 11 different species and found species- and RNA biotype-specific features of divergent/convergent transcription. Furthermore, we confirmed that CCIVR2 enables the identification of multiple sense/antisense transcript pairs from divergent transcription, including those with known functions in processes such as embryonic stem cell differentiation and TGFβ stimulation. CCIVR2 is therefore a valuable bioinformatics tool that facilitates the characterization of divergent/convergent transcription in different species and aids the identification of functional sense/antisense transcript pairs from divergent transcription in specified biological processes.

## Introduction

A gene corresponds to a single transcription unit starting from a promoter, which may or may not contain TATA elements. In promoters with TATA elements, the TBP complex binds to the TATA element in a direction-dependent manner, thereby determining unidirectional transcription. In contrast, in promoters with CpG-rich elements instead of TATA elements, the TBP complex binds in both directions via CpG-binding transcription factors, such as Sp1, resulting in bidirectional transcription^1^. Most mammalian promoters lack TATA elements but are CpG rich. Consistently, many instances of bidirectional transcription have been identified in mammals^2, 3^. Bidirectional transcription is known as divergent transcription^3–5^, which typically generates short (50–2000 nucleotide) and relatively unstable upstream antisense RNAs, termed transcription start site-associated (TSSa)-RNAs, upstream antisense RNAs (uaRNAs), and PROMoter uPstream Transcripts (PROMPTs)^4–7^. Interestingly, this divergent transcription is hypothesized to be a driving force for the generation of new genes. In this model, initially short and unstable TSSa-RNA/uaRNAs/PROMPTs, evolve into longer and more stable genes by gaining U1 sites and losing polyadenylation signals (PASs)^8^. Indeed, some transcripts from divergent transcription are a protein-coding mRNA and a long non-coding RNA (lncRNA) that contain relatively long exon/intron structures and polyA sequences^9, 10^, and some of these lncRNAs function to regulate gene expression^11, 12^. However, the functions of many transcripts from divergent transcription are largely unknown.

The single transcription unit of a gene terminates at the terminator. Termination of transcription by eukaryotic RNA polymerase II (Pol II) proceeds in a stepwise manner. The largest subunit of Pol II contains a unique carboxyl-terminal domain (CTD) consisting of a repeated heptameric sequence (YSPTSPS). During transcription elongation, the second serine in the heptad repeat is phosphorylated and this enhances interaction with the 3′-end cleavage and polyadenylation (CPA) complex^13, 14^. Among alternative polyA sites, a functional PAS is sensed by CPA factors causing slowing of Pol II transcription^15^. The transcribed PAS is cleaved and a polyA sequence is added by CPA factors to generate the mRNA end sequence, called the transcript end site (TES). Transcription continues through the PAS and is completed by structural changes in Pol II (allosteric model)^16^ and/or degradation of the co-transcriptionally cleaved transcript (CoTC sequence) by Xrn2, which causes Pol II to detach from the RNA and terminate transcription (torpedo model)^17, 18^. This detaching point of Pol II is called the transcription termination site (TTS) and is usually located several kilobases after the PAS, although some transcripts terminate shortly after the PAS. Pairs of transcription consisting of one sense transcription and one antisense transcription that are initiated from different gene regions and overlap in the 3′ region are called convergent transcription. The overlap region at the 3′ end of sense/antisense pairs can function in regulating the expression of partner genes through polymerase collisions and double-strand RNA formation, which can mask the binding of miRNAs^19, 20^. Although convergent transcription and the mechanism of its regulation have been reported, structural characteristics of convergent transcription in different species or RNA biotypes are poorly defined.

Natural antisense transcripts (NATs) encode sequences that are complementary to RNA transcripts of genes. Unlike trans-NATs, whose partner genes are other genomic loci, cis-NATs are transcribed from the opposite strand of their partner genes and overlap completely or partially with their partner genes. Cis-NATs can be classified into four different types according to their structural characteristics. In the embedded type (EB), the entire transcription unit of the antisense gene is embedded in the transcription unit of the sense gene. In the fully overlapped type (FO), the antisense transcription unit covers the entire sense gene. In the head-to-head (HH) type, the sense and antisense genes partially overlap only at their 5′ ends, while in the tail-to-tail (TT) type the genes partially overlap only at their 3′ ends. We previously developed a simple and convenient program termed CCIVR (*c*omprehensive *c*is-NATs *i*dentifier *v*ia *R*NA-seq data) that can identify cis-NATs with their structural features including EB, FO, HH, and TT, based on locational information^21^. However, CCIVR can only identify overlapping antisense transcripts and not non-overlapping transcripts, such as nearby head-to-head (nHH) and nearby tail-to-tail (nTT) transcripts, which may function in collaboration with their partner genes.

Here, we describe CCIVR2, an extension of our CCIVR program, which facilitates the identification of all types of cis-NAT, as well as 5′ (i.e. nHH) and 3′ (i.e. nTT) non-overlapping antisense transcripts. Furthermore, in CCIVR2, the user can specify the region where antisense transcription start sites (TSSs) or transcript end sites (TESs) are located to identify both overlapping and non-overlapping antisense transcripts within the region. CCIVR2 can comprehensively identify antisense transcripts from divergent transcription, including those with known functions. CCIVR2 therefore facilitates the characterization of TSSs and TESs in sense/antisense gene pairs and aids the identification of candidate functional antisense transcripts from divergent transcription.

## Results

### Overview of CCIVR2 and its operating principles

**Figure 1:**
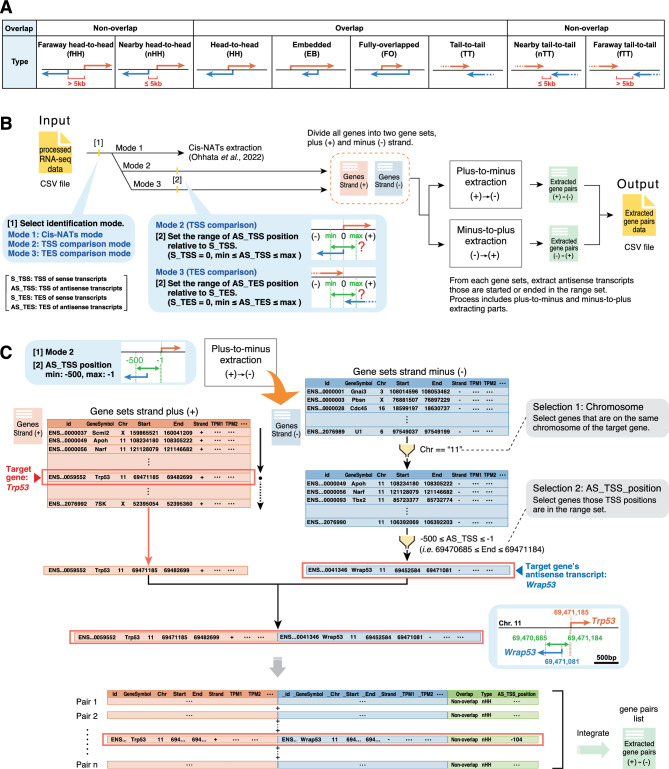
An overview of the CCIVR2 processing steps. (**A**) Definition of the types of antisense transcript analyzed in this study. CCIVR2 can extract eight types of antisense transcript; four types of overlapping antisense transcript (cis-NATs: HH, EB, FO, and TT) and four types of non-overlapping antisense transcript (fHH, nHH, fTT, and nTT). Among the non-overlapping antisense transcripts, those within 5 kb are defined as "nearby" and those further apart are defined as "faraway". (**B**) Overview of CCIVR2. CCIVR2 contains three different modes; Mode 2 and 3 are newly implemented functions that can extract non-overlapping antisense transcripts within a specified region. After selecting Mode 2/3, users specify two parameters (min, max) to define the range within which the antisense transcripts will be identified. For details, please see the README.md file placed at https://github.com/CCIVR/ccivr2. (**C**) An example of the process for a target gene, *Trp53*, and the identification of its non-overlapping antisense transcript, *Wrap53*. The process focuses on the plus-to-minus extraction part in Mode 2 (TSS comparison) to extract the antisense transcripts whose TSSs are between −500 and −1 bp relative to the TSS of the sense transcript.

We previously developed a program termed CCIVR that enables the identification of cis-NATs according to their structural features^21^; however, CCIVR can only identify overlapping and not non-overlapping antisense transcripts, which may be functional. To identify and investigate antisense transcripts that do not overlap with the sense transcript but that are transcribed nearby and faraway, we developed CCIVR2. We distinguished non-overlapping antisense transcripts adjacent to a sense transcript (within 5 kb) as “nearby” and those more distant as “faraway” (Fig. 1A). Accordingly, CCIVR2 can extract eight types of antisense transcript: four types of overlapping cis-NAT (HH, EB, FO, and TT) and four types of non-overlapping antisense transcript [faraway head-to-head (fHH), nearby head-to-head (nHH), faraway tail-to-tail (fTT), and nearby tail-to-tail (nTT)] (Fig. 1A).

An overview of CCIVR2 is shown in Fig. 1B. The input file for CCIVR2 analysis contains locational information for each gene from the Ensembl database, including chromosome location, strand direction, TSS and TES, as well as expression profile data, such as TPM (Transcripts Per Million), obtained from processed RNA-seq data using a peer-reviewed tool such as RSEM^22^ (for details, please see the README.md file at https://github.com/CCIVR/ccivr2). CCIVR2 can be run in one of three modes (Fig. 1B). Mode 1 is the same as the previous CCIVR, which comprehensively extracts four types of cis-NAT^21^, whereas Modes 2 and 3 extract non-overlapping antisense transcripts within a specified region. Mode 2, or TSS comparison mode, can comprehensively isolate antisense transcripts whose TSSs are within a set range of positions relative to the TSSs of the sense transcripts, while Mode 3, or TES comparison mode, can comprehensively isolate antisense transcripts whose TESs are within a set range of positions relative to the TESs of the sense transcripts. The range can be set in both minus (i.e. upstream) and plus (i.e. downstream) positions relative to the TSSs/TESs of the sense transcripts. Therefore, non-overlapping antisense transcripts can be extracted when the range is set in the minus position (i.e. upstream) in Mode 2 and in the plus position (i.e. downstream) in Mode 3. Conversely, if the range is set to the plus position in Mode 2 or to the minus position in Mode 3, it is also possible to extract the overlapping cis-NATs as well as the non-overlapping antisense transcripts beyond them. After setting the range, the input data is divided into two groups of gene sets according to whether the genes are on the plus ( +) or minus (−) strand. Then, the antisense transcripts that match the conditions are sequentially extracted from each strand in two parts: plus-to-minus extraction and minus-to-plus extraction. The antisense transcripts extracted in each part are listed as gene pairs in a transient dataset. Finally, these two transient datasets are combined to generate an output file that contains all antisense transcript data (Fig. 1B).

As an example, plus-to-minus extraction with Mode 2 selected and the extraction range set to (min: −500, max: −1) is shown in Fig. 1C. A mouse dataset that contains 55,414 genes (Ensembl GRCm39.105) was subjected to the process. The *Trp53* gene, which encodes the tumor suppressor protein p53, was chosen as an example of a target gene on the plus strand, while the *Wrap53* gene, which encodes an antisense transcript of *Trp53* that positively regulates p53 mRNA and protein levels in human cells^23^, was chosen as an example of an extracted nHH antisense transcript on the minus strand. The *Trp53* gene is located on chromosome 11; therefore, only genes on the minus strand of the same chromosome were filtered (Selection 1: Chromosome, Fig. 1C). The filtered genes were then screened for nHH criteria of the *Trp53* gene [condition: 69,470,685 ≤ End ≤ 69,471,184] (Selection 2: AS_TSS_position, Fig. 1C). *Trp53* and *Wrap53* information were combined as paired data attached to their structural features. All gene pairs matching the conditions were then extracted and integrated to complete the transient dataset "Extracted gene pairs plus-to-minus". In this way, CCIVR2 enables comprehensive identification of sense/antisense pairs that meet the set of extraction conditions by comparing the detailed positional information of each gene.

### Identification of divergent and convergent transcription in representative model organisms

We next attempted to comprehensively extract antisense genes with TSSs within 5 kb upstream to 5 kb downstream of the TSS of a sense gene (i.e. divergent transcription, including both overlapping and non-overlapping). Similarly, we also attempted to comprehensively extract antisense genes with TESs within 5 kb upstream to 5 kb downstream of the TES of a sense gene (i.e. convergent transcription including both overlapping and non-overlapping) (please note that under the conditions of this divergent transcription analysis, all antisense genes transcribed upstream of the TSS of the sense gene are of the nHH type, whereas antisense genes transcribed downstream of the TSS of the sense gene can be of the HH, EB, FO, TT, and nTT types. These features also apply to the convergent transcription analysis. These analyses are made possible by the new specifications of CCIVR2, which were not possible with our previous program, CCIVR^21^). As in previous CCIVR analyses^21^, we chose 11 genetically well-studied representative model organisms for CCIVR2 analysis. Each species has various types of RNA (Fig. S1). Therefore, we first analyzed the data by using all types of RNA (Fig. S2). The regions to be analyzed comprised 20 sections of 500 bp in the interval from 5 kb upstream to 5 kb downstream, and the percentage of genes possessing antisense transcripts to the total number of genes in each particular region was calculated.

**Figure 2:**
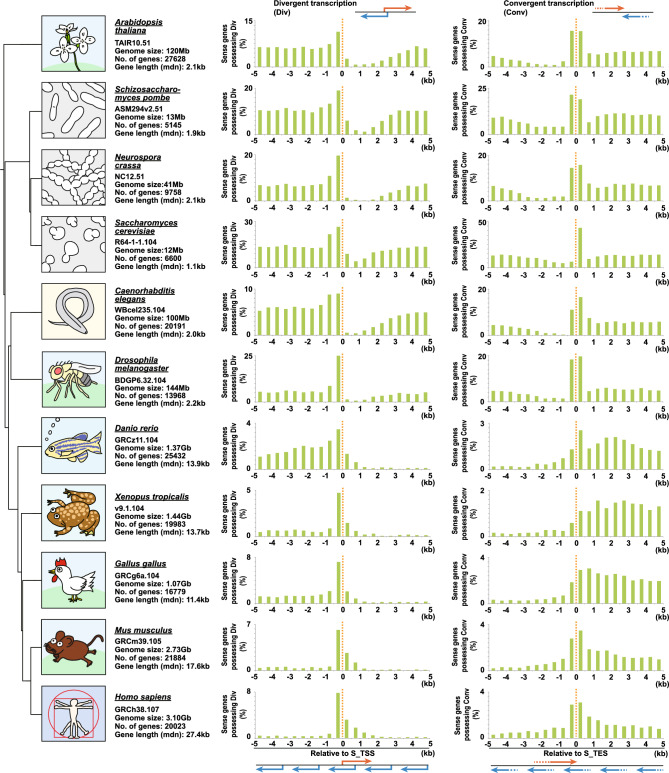
Identification of divergent and convergent transcription within the specified region: protein-coding genes from representative model organisms. The percentage of sense transcripts that possess a TSS or TES of an antisense transcript in the specified region relative to the TSS or TES of the sense transcripts is shown. The range of each specified region is 500 bp. The results of the protein-coding genes from 11 different model organisms are shown. Red and blue arrows represent sense transcripts and their antisense transcripts, respectively. The genome data version from Ensembl/Ensembl_plant/Ensembl_fungi, genome size, number of genes, and median (mdn) gene length are shown below the name of each species. The phylogenetic tree was generated using phyloT_v2 (https://phylot.biobyte.de).

The results showed that the presence of sense/antisense pairs tended to be higher near the TSS/TSS and TES/TES contact points for both divergent and convergent transcription analysis (Fig. S2). However, for some species the trend was less remarkable. For example, in *Caenorhabditis elegans*, we observed a high prevalence of sense/antisense pairs across the entire analyzed range (Fig. S2), likely because of the presence of large amounts of piRNAs specific to the species (Fig. S1). To avoid the influence of species-specific RNA types, we performed a series of such analyses focusing on protein-coding genes only. The results confirmed divergent and convergent transcription to have the trend of protein-coding genes having a high percentage of sense/antisense pairs around their TSS/TSS and TES/TES contact points (Fig. 2). This may indicate that they share their promoters and polyA signals. Indeed, two pairs of convergent transcription share their polyA signals in *Arabidopsis thaliana*^24^. However, we cannot rule out the possibility that the adjacency of genes is simply a stochastic increase in those species whose genomes and gene lengths are relatively short, such as *A. thaliana*, *Schizosaccharomyces pombe*, *Neurospora Crassa*, *Saccharomyces Cerevisiae*, *C. elegans*, and *Drosophila melanogaster*.

In these species, whose genomes and gene lengths are relatively short, the abundance of antisense TSS-harboring genes decreased significantly in the regions downstream of sense genes’ TSSs (i.e. 5' end of the gene body of the sense genes) (Fig. 2, divergent transcription). In contrast, the abundance of antisense TES-harboring genes decreased significantly in the regions upstream of sense genes’ TESs (i.e. 3' end of the gene body of the sense genes) (Fig. 2, convergent transcription). We speculate that these trends observed in these species reflect the avoidance of duplication at a gene location. To investigate whether this trend is applicable to other species with relatively long genomes and gene lengths, such as *Danio rerio*, *Xenopus tropicalis*, *Gallus gallus*, *Mus musculus*, and *Homo sapiens*, we expanded the range of analysis to reflect the gene length of each species. We found that the trend was observed in all species (Fig. S3). This indicates that the trend to avoid duplication of antisense transcription within the gene body of a protein-coding gene is common to many species. Therefore, CCIVR2 facilitates analysis of transcription start and end point characteristics of sense/antisense pairs based on gene sequence information.

### Comparison of divergent and convergent transcription among different RNA biotypes

**Figure 3:**
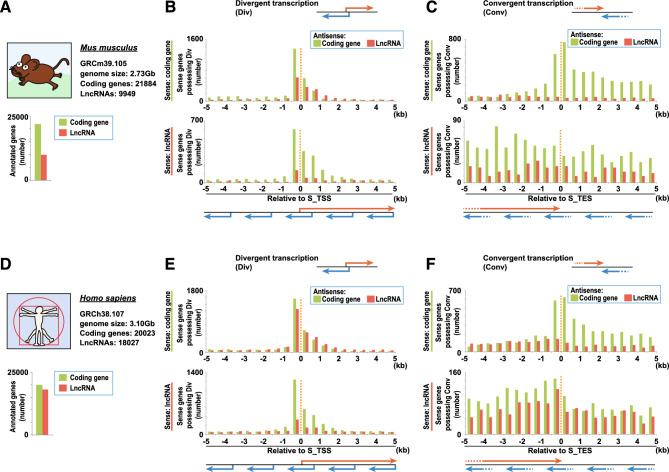
Comparison of divergent and convergent transcription between coding genes and lncRNAs in mouse and human. (**A**–**C**) Identification of divergent and convergent transcription with protein-coding genes and lncRNAs in mouse. The Ensembl database version used in this study, the genome size, and the number of coding genes and lncRNAs annotated in the database are shown below the name of the species (**A**), and the number of sense transcripts that possess a TSS (**B**) or TES (**C**) of an antisense transcript in the specified region relative to the TSS (**B**) or TES (**C**) of the sense transcripts is shown. Their gene biotypes (i.e. coding gene or lncRNA) are specified in each panel. The width of each specified region is 500 bp. Red and blue arrows represent sense transcripts and their antisense transcripts, respectively. (**D**–**F**) Identification of divergent and convergent transcription with protein-coding genes and lncRNAs in human, as shown in (**A**–**C**).

CCIVR2 analysis revealed that the sharing of TSSs and TESs by protein-coding gene sense/antisense transcript pairs is relatively common in some species. To investigate whether this feature is also applicable to other RNA types, we first focused on lncRNAs and performed divergent transcription analysis using two species, *M. musculus* and *H. sapiens*, in which large numbers of lncRNAs have been reported (Fig. S1, 3A, and 3﻿D). Analysis of murine divergent transcription showed highly comparable patterns of distribution between cases in which the sense strand was a coding gene and the antisense strand was an lncRNA and cases in which both the sense and antisense strands were coding genes (Fig. 3B). Furthermore, their ratio was comparable to those of lncRNAs and coding genes as a whole [Fig. 3A,B, ratio of lncRNA/coding and coding/coding in the divergent transcription highest peak (−500, −1), 606/1347, 45.0%; ratio of lncRNA/coding as a whole, 9949/21,884, 45.5%]. This feature was also confirmed by divergent transcription analysis in human [Fig. 3D,E, ratio of lncRNA/coding and coding/coding in the divergent transcription highest peak (−500, −1), 1262/1564, 80.7%; ratio of lncRNA/coding as a whole, 18,027/20,023, 90.0%]. However, in the murine analysis, the patterns were similar for cases in which both the sense and antisense strand were lncRNAs and for cases in which the sense strand was an lncRNA and the antisense strand was a coding gene, but their ratio was lower than the ratio of lncRNAs and coding genes as a whole [Fig. 3A,B, ratio of lncRNA/lncRNA and lncRNA/coding in the divergent transcription highest peak (−500, −1), 140/602, 23.3%; ratio of lncRNA/coding as a whole, 9949/21,884, 45.5%]. This feature was also confirmed by divergent transcription analysis in human [Fig. 3D,E, ratio of lncRNA/lncRNA and lncRNA/coding in the divergent transcription highest peak (−500, −1), 335/1238, 27.1%; ratio of lncRNA/coding as a whole, 18,027/20,023, 90.0%]. In contrast, analysis of divergent transcription with pseudogenes showed that the number of pseudogene/coding and pseudogene/pseudogene pairs was greatly reduced in both mouse (Fig. S4A and S4B) and human (Fig. S4D and S4E). Taken together, CCIVR2 revealed RNA biotype-specific features of sense/antisense transcript pairs that share their TSSs in mouse and human and that coding/coding and lncRNA/coding pairs are equally abundant. In contrast, lncRNA/lncRNA, pseudogene/coding, and pseudogene/pseudogene pairs were rarely identified.

We then performed a convergent transcription analysis to investigate whether sharing TESs is applicable to RNA biotypes other than protein-coding genes. The lncRNA analysis showed no tendency of TESs to be shared by lncRNA/coding and lncRNA/lncRNA pairs, either in mouse (Fig. 3C) or human (Fig. 3F). Moreover, the pseudogene analysis also showed no tendency of TESs to be shared by pseudogene/coding and pseudogene/pseudogene pairs, either in mouse (Fig. S4C) or human (Fig. S4F). Long intergenic non-coding RNAs (lincRNAs), a subset of lncRNAs, are mainly non-polyadenylated^25^. However, the mouse and human lncRNA data in the Ensembl database that we used in this study are based on HAVANA annotations, and most entries have experimentally confirmed polyA signals, polyA tags, or polyA sequences. Therefore, the reason that lncRNAs do not share their TESs is not because of an absence of polyA features. Taken together, CCIVR2 revealed the RNA biotype-specific features of sense/antisense transcript pairs that share their TESs in mouse and human. Coding/coding pairs were abundant but pairs with lncRNAs and pseudogenes were rarely identified.

### Identification of divergent transcription during specific processes

**Figure 4:**
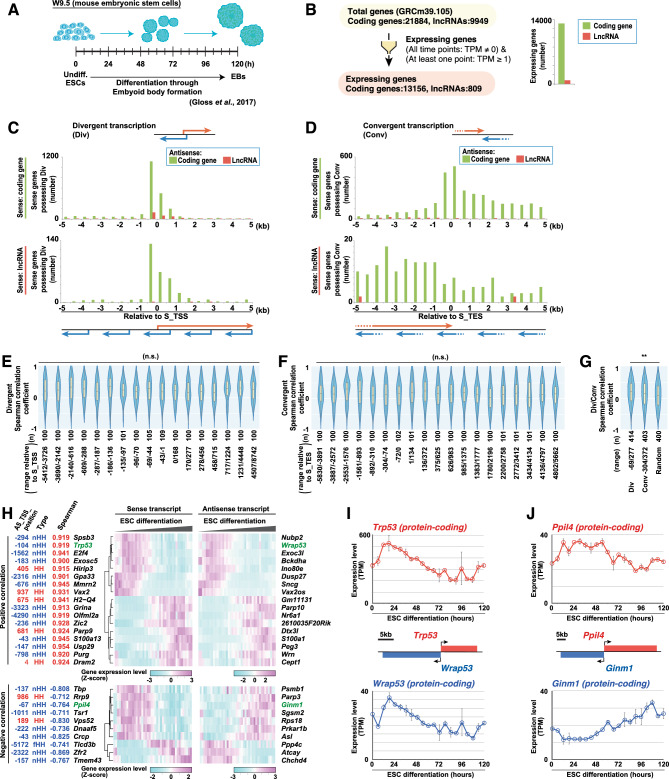
Example of CCIVR2 analysis I: identification of divergent transcription upon ESC differentiation. (**A**) Schematic representation of the procedure for differentiating ESCs through embryoid body formation^26^. (**B**) Selection of expressing genes upon ESC differentiation. The definition and number of expressing genes are shown. (**C**,**D**) Identification of divergent (**C**) and convergent (**D**) transcription from expressing genes upon ESC differentiation, as shown in Fig. 3. (**E**–**G**) Spearman correlation coefficient analysis, comparing the gene expression dynamics between sense and antisense transcripts upon ESC differentiation. Box-and-whisker plots are shown within the violin plots for the specified regions of divergent (**E**), convergent (**F**), and divergent/convergent (**G**) transcription. n.s.: not significant, **p < 0.01, Steel–Dwass’s multiple comparison test. (**H**) Heatmap analysis of sense/antisense transcript pairs from divergent transcription. Positively and negatively correlating genes were selected when the Spearman correlation coefficient was greater than 0.9 and less than − 0.7, respectively. The distance between the sense and antisense TSSs of the transcripts from divergent transcription is shown (AS_TSS_position). The genes in green font were chosen for the further studies. (**I**,**J**) Expression dynamics of *Trp53*/*Wrap53* (**I**) and *Ppil4*/*Ginm1* (**J**) sense/antisense transcript pairs upon ESC differentiation. Means ± SD across biological replicates (TPM from RNA-seq, n = 2) are shown.

Finally, we assessed whether CCIVR2 can identify overlapping/non-overlapping antisense transcripts that function in specific processes. We chose datasets of mouse embryonic stem cell (ESC) differentiation via embryoid body (EB) formation (Fig. 4A)^26^ and epithelial-mesenchymal transition (EMT) of Huh-7 human liver cancer cells in response to TGFβ (Fig. S5A)^21, 27^. Based on RNA-seq data, we defined “expressing genes” among both coding genes and lncRNAs as those with a TPM greater than 1 at least one of the time points and not 0 at any time point (Fig. 4B and Fig. S5B). We performed divergent transcription analysis of the expressing genes and found that the distribution patterns were highly comparable between cases where both the sense and antisense strands were coding genes (coding/coding) and cases where the sense strand was a coding gene and the antisense strand was an lncRNA (coding/lncRNA). This was observed in both differentiating ESCs (Fig. 4C) and TGFβ-responding Huh-7 cells (Fig. S5C), and the distribution pattern was consistent with the results for all annotated genes in mouse (Fig. 3B) and human (Fig. 3E). We then performed the convergent transcription analysis with the expressing genes and found that the convergent pairs with neighboring TESs were abundant among coding/coding pairs, but not with lncRNAs. This was observed in both differentiating ESCs (Fig. 4D) and TGFβ-responding Huh-7 cells (Fig. S5D), and is consistent with the results for all annotated genes in mouse (Fig. 3C) and human (Fig. 3F). Therefore, the features of divergent and convergent transcription observed in annotated genes were confirmed in genes expressed in specific processes, such as ESC differentiation and the TGFβ response.

We then investigated whether the correlation of the expression pattern of each gene from a sense/antisense pair varies with the distance between their TSSs and TESs by performing Spearman correlation coefficient analysis using RNA-seq samples of differentiating mouse ESCs. For the analysis, both protein-coding and lncRNA expressing genes were used (Fig. 4B). We extracted bins of 100 sense/antisense pairs (note: in some regions, the number may exceed 100 because of the presence of multiple pairs at the edge of the region) in the 5 kb upstream and downstream relative to the sense TSS/TES, and examined the correlation of these expression patterns in divergent (Fig. 4E) and convergent (Fig. 4F) transcription. The results showed no significant differences in correlation (Fig. 4E,F). However, comparisons of 400 pairs generated around the sense TSS in divergent transcription, around the sense TES in convergent transcription, and randomly generated pairs on the same chromosome showed a significant positive correlation in divergent transcription compared with randomly generated pairs (Fig. 4G). These trends were also confirmed by analysis of TGFβ-responding Huh-7 cells (Fig. S5E–G). These results indicate that the number of antisense transcripts is increased near the sense TSS, but that the quality (i.e. the pattern of gene expression) is not affected within 5 kb upstream or downstream of the sense TSS.

Finally, we attempted to identify functional overlapping/non-overlapping transcripts from divergent transcription that showed positive and negative correlation from the differentiating ESC dataset. Among the sense/antisense transcript pairs whose correlation was analyzed, the Spearman correlation coefficients greater than 0.9 and less than − 0.7 were defined as positive and negative correlations, respectively. The gene expression patterns were then displayed as a heatmap, along with types of antisense transcript (Fig. 1A) and the position of the antisense TSS relative to the sense TSS (Fig. 4H). In the list, we focused on the pairs *Trp53*/*Wrap53* and *Ppil4/Ginm1* as representative non-overlapping sense/antisense transcript pairs from divergent transcription whose expression was positively or negatively correlated, respectively. *Wrap53* is an antisense-oriented transcript of *Trp53*, which encodes the tumor suppressor protein p53. *Wrap53* enhances the level of p53 mRNA and protein in human cells^23^. Consistent with this, we found their coordinated expression was synchronously up-regulated and then down-regulated upon ESC differentiation (Fig. 4I). *Ppil4*, which encodes peptidyl-prolyl c*is–trans* isomerase-like 4, is essential for vertebrate brain angiogenesis^28^. Upon ESC differentiation, a change in *Ppil4* expression was observed; it initially increased, maintained the higher level of expression, and finally decreased (Fig. 4J). *Ginm1*, which encodes glycosylated integral membrane protein 1 (of unknown function), is transcribed from upstream of the *Ppil4* promoter, and its expression was negatively correlated with the expression of *Ppil4* (Fig. 4J). The mechanism of the reciprocal regulation of gene expression between *Ppil4* and *Ginm1* remains to be elucidated. Likewise, functionally-annotated non-overlapping antisense transcripts from divergent transcription could be identified from the TGFβ-responding Huh-7 cell dataset (Fig. S5H). This included the lncRNA/coding pair, *PCAT6*/*KDM5B*, and the coding/lncRNA pair, *LHX1*/*LHX1-DT*, which functions in malignant tumor cell growth. *PCAT6* enhances proliferation and EMT in gastric cancer (GC)^29^, and *KDM5B* enhances EMT in lung cancer cell line, A549^30^, and in hepatocellular carcinoma^31^. *LHX1* is involved in modulating EMT induction in uterine corpus endometrial carcinoma^32^, and *LHX1-DT* enhances cell proliferation in breast cancer^33^. These coordinated gene expression changes that were up-regulated by TGFβ stimulation were also confirmed by qRT-PCR (Fig. S5I and S5J). In addition to these sense/antisense transcript pairs from divergent transcription, some of which have been reported their functions and regulation of expression, many others with unknown functions were identified (Fig. 4H and S5H) and we predict that some of them will have functions. Among the candidates extracted under these conditions, 20 out of 27 (Fig. 4H) and 15 out of 33 (Fig. S5H) were non-overlapping antisense transcripts from divergent transcription that were isolated for the first time by the new specifications of CCIVR2. Therefore, CCIVR2 facilitates obtaining a comprehensive set of candidate transcripts from divergent transcription that may be functionally involved in regulating the expression of partner genes or possess functions in specified processes.

## Discussion

In this study, we developed a program called CCIVR2, which is an extension of our previously reported program, CCIVR^21^. CCIVR2 has two major improvements compared with CCIVR. First, it can set the range of sense/antisense pairs to be extracted, the range of antisense TSSs relative to sense TSSs, and the range of antisense TESs relative to sense TESs. Second, it facilitates the extraction of not only overlapping sense/antisense pairs, but also non-overlapping sense/antisense pairs by type (Fig. 1A). The limitations of CCIVR2 are as follows: (1) the transcript data for CCIVR2 analysis are annotated but not de novo assembled; therefore, novel transcripts may be missed. (2) CCIVR2 only recognizes the TSSs/TESs of genes but not their exon/intron structure; therefore it does not distinguish whether the overlapping region contains exon sequences. (3) The CCIVR2 process depends on third-party programs, such as STAR^34^ for mapping, RSEM^22^ for expression profiling, and DESeq2^35^ for statistical analysis; i.e. CCIVR2 scripts only cover part of the full CCIVR2 analysis pipeline. In addition, many bioinformatics tools can identify or analyze cis-NATs and/or divergent/convergent transcription. For example, NASTI-seq^36^ allows reliable detection of cis-NATs using the variable error rate of a strand-specific protocol, NATpipe^37^ allows systematic discovery of NATs from de novo assembled transcriptomes, BEDTools^38^ allows identification of overlapping cis-NAT pairs based on genome annotation, Tfit^39^ allows identification of sites of divergent transcription in nascent transcription assays such as Global Run-On (GRO) and Precision Run-On (PRO) sequencing data, and *GenomicRanges*^40^ allows detection of overlapping and neighboring genes from annotated genomic information. While these tools provide reliable methods for such analyses, CCIVR2 has the unique feature of being able to comprehensively extract sense/antisense pairs along with annotation of the type of antisense transcript within a specified range. This feature of CCIVR2 allows structural analysis of divergent/convergent transcription and screening for functional antisense transcripts in specified processes.

In this study, we analyzed divergent transcription using CCIVR2. In mammals, relatively unstable and short transcripts without a polyA tail, called TSSa-RNAs/uaRNAs/PROMPTs, are transcribed in the opposite direction to protein-coding genes from promoters with CpG rich elements. It is hypothesized that these expressed loci have evolved into lncRNAs and protein-coding genes over a long period of time^8^. Indeed, we observed many protein-coding genes and lncRNAs in divergent pairs (Fig. 3), whereas pseudogenes, which are considered to be descendants rather than ancestors of protein-coding genes^41^, were rare in divergent pairs with protein-coding genes (Fig. S4). This hypothesis is also supported by the low number of lncRNA/lncRNA divergent pairs we observed (Fig. 3). Furthermore, these characteristics were confirmed in gene groups for which annotations were registered and also in gene groups for which gene expression was confirmed in specific processes, such as ESC differentiation (Fig. 4C) and TGFβ stimulation (Fig. S5C). Further investigation is required to confirm genomic sequence characteristics, such as the presence of CpG rather than TATA elements. We also comprehensively identified sense/antisense transcript pairs from divergent transcription, changes in which were positively and negatively correlated with specific processes (Figs. 4H and S5H). In some cases, both sense and antisense transcripts were functionally involved in the process (*e.g. PCAT6*/*KDM5B* both induce EMT). It is not known whether the positively correlated gene expression is merely the result of the same regulation for both genes or whether there is a positive feedback regulation mechanism between them. Further investigations are required to clarify the mechanism of expression regulation between divergently transcribed gene pairs by suppressing the functions of RNAs using approaches such as morpholino antisense oligonucleotides (mASOs), RNA interference (RNAi), and clustered regularly interspaced short palindromic repeats (CRISPR)/Cas7-11^42^ and by controlling the expression of target genes using CRISPR-mediated transcriptional interference/CRISPR-mediated transcriptional activation (CRISPRi/CRISPRa)^43^.

In this study, we also analyzed convergent transcription using CCIVR2. We found that the number of convergently transcribed gene pairs whose TESs are proximately located is relatively increased in mouse and human (Fig. 2). This is pronounced for coding/coding pairs, but is not the case when lncRNAs or pseudogenes are paired with protein-coding genes (Figs. 3 and S4). Therefore, protein-coding genes of mice and humans may have peculiarities that cause their TESs to be located in close proximity to each other. The possibility that this feature is caused by artificial cDNA synthesis, such as from AT rich sequences, cannot be ruled out. However, we consider this to be unlikely because the 3′ annotation of the Ensembl genes includes not only the polyA sequence but also the presence of the polyA signal. Furthermore, this feature is only observed for protein-coding pairs and not for lncRNAs or pseudogenes, supporting exclusion of this possibility. CCIVR2 analysis uses a representative form of gene structure, which is selected from a gene’s variants. Although the longest TES is chosen as the representative TES for genes registered in the Ensembl database, there are multiple polyA sites and TESs for each gene. Therefore, if multiple TESs of each gene could also be analyzed, the number of convergent gene pairs with adjacent TESs might be further increased. Transcription can be completed by structural changes in Pol II (allosteric model)^16^ and by degradation of the cotranscriptionally cleaved transcript by Xrn2, which causes Pol II to detach from the RNA and terminate transcription (torpedo model)^17, 18^. In addition, R-loops can form at G-rich terminator elements and promote RNA interference-dependent formation of a repressive chromatin environment, such as the repressive histone mark H3K9me2, which reinforces Pol II pausing and transcription termination in mammalian protein-coding genes^44^. Therefore, the mechanism of transcription termination on the sense strand may affect the transcription termination on the antisense strand through formation of a repressive chromatin environment. In general, many Pol II-transcribed genes exhibit gradual termination profiles over several kilobases after the PAS, while others terminate immediately after the PAS^15^. Therefore, in addition to sharing the PAS, the transcription termination mechanism may also be involved in selecting the TESs of convergent transcript pairs. A long read-through after a TES can affect the expression of neighboring genes, for example because of Pol II collision. Our findings may indicate a mechanism to avoid this, and further molecular investigations focusing on individual sense/antisense transcript pairs sharing their TESs are warranted.

Here, we have introduced an original program, CCIVR2, that facilitates the analysis of overlapping and non-overlapping antisense transcripts as well as divergent/convergent transcription. We believe that CCIVR2 will drive the study of overlapping and non-overlapping antisense transcripts to elucidate their mechanisms of action and functions in numerous biological processes in various species.

## Materials and methods

### CCIVR2 analysis with annotated genes

GTF files from 11 species were downloaded from Ensembl plant (https://plants.ensembl.org/index.html; *Arabidopsis thaliana*: TAIR10.51), Ensembl Fungi (https://fungi.ensembl.org/index.html; *Schizosaccharomyces pombe*: ASM294v2.51, *Neurospora crassa*: NC12.51, *Saccharomyces cerevisiae*: R64-1-1.104), and Ensembl (https://www.ensembl.org/index.html; *Caenorhabditis elegans*: WBcel235.104, *Drosophila melanogaster*: BDGP6.32.104, *Danio rerio*: GRCz11.104, *Xenopus tropicalis*: v9.1.104, *Gallus gallus*: GRCg6a.104, *Mus musculus*: GRCm39.105, *Homo sapiens*: GRCh38.107). Only the gene information listed in the feature column of the GTF files was extracted and converted to a CSV file using Python (ver 3.8.8) and one of its modules, gtfparse (ver 1.2.1). To avoid the analysis of duplicate genes, only The European Nucleotide Archive (ENA) for *N. crassa* and PomBase for *S. pombe* were used as their gene_source. For the other species, every gene_source was used for CCIVR2 analysis because no duplication was observed. The number of genes was counted from gene_id and not GeneSymbol because we found that every gene was unique in gene_id but that this was not the case for some genes in GeneSymbol. The CSV formatted input files for the CCIVR2 analysis, containing all RNA biotypes or only protein-coding genes, were applied to a python script "autoccivr2.py", which allows both Mode 2 and Mode 3 of the CCIVR2 analysis to be performed with different ranges on multiple datasets simultaneously. The number of antisense pairs with different RNA biotype combinations, such as with lncRNAs and pseudogenes, was counted using Python scripts "count_cg_lr.py" and "count_cg_pg.py", respectively. The phylogenetic tree was generated using phyloT-v2 (https://phylot.biobyte.de)^45^.

### RNA-seq analysis

The SRA files for ESC differentiation (accession number: GSE07528)^26^ were downloaded from The National Center for Biotechnology Information (NCBI). The SRA files for the TGFβ response in Huh-7 cells (accession number: DRA01352)^21, 27^ were downloaded from the DNA Data Bank of Japan (DDBJ). Low-quality RNA-seq reads and adaptor sequences were removed using Trim Galore!. Sequence reads were aligned to the mouse reference genome (GRCm39.105) or human reference genome (GRCh38.107) using STAR (version 2.7.9a)^34^ allowing up to three mismatches. For each gene, TPM was calculated using RSEM (version 1.3.3)^22^. Differential expression analysis (Wald test) was performed using DESeq2^35^. The expressing genes were sorted using the Excel sorting function, with the criteria that the TPM was greater than 1 at least one of the time points and not 0 at any time point.

### CCIVR2 analysis of expressing genes

The CSV formatted input files containing expressing genes with their TPM and statistical analysis (i.e. padj) were applied to a Python script "autoccivr2.py", and the number of antisense pairs with different RNA biotype combinations, such as protein-coding genes and lncRNAs, was counted using a Python script "count_cg_lr.py". The input files were subjected to CCIVR2 analysis with the following conditions: (Mode 2; min: −10,000; max: −10,000). Spearman correlation coefficient analysis between the sense and antisense transcripts was performed using the Excel functions combined with "RANK" and "CORREL". Bins of 100 sense/antisense pairs (depending on the region, the number may exceed 100 because of the presence of multiple pairs at the edge of the region) in the region 5 kb upstream and downstream relative to the sense TSS/TES were identified and visualized by a violin plot using a Python method "violin" from one of its packages, plotly (ver 5.11.0). The Steel–Dwass multiple comparison test was performed using a Python method "posthoc_dscf" from one of its packages, scikit_posthocs (ver 0.7.0). Heatmaps for selected positively and negatively correlated genes were generated using R (ver 4.1.1) with "*gplots*" (ver 3.1.1) function.

### Cell culture and reagents

Human hepatoma cell line Huh-7 (JCRB0403) was purchased from JCRB Cell Bank (National Institute of Biomedical Innovation, Osaka, Japan), grown in Dulbecco’s modified Eagle’s medium (Sigma-Aldrich, St. Louis, MO, USA) supplemented with 10% fetal bovine serum (Sigma-Aldrich) and 1 × penicillin/streptomycin (Meiji Seika Pharma Co., Ltd., Tokyo, Japan) at 37 °C under an atmosphere containing 5% CO_2_. Recombinant hTGFβ1 (240-B; R&D systems, Minneapolis, MN, USA) was added to a final concentration of 10 ng/ml for TGFβ stimulation.

### RT-qPCR

Total RNA was purified using an RNeasy Mini Kit (Qiagen). For RT-qPCR, cDNA was prepared using SuperScript II reverse transcriptase (Invitrogen) with random primers (Invitrogen). RT-qPCRs were performed in duplicate using Thunderbird SYBR qPCR mix (Toyobo, Osaka, Japan) with the primers listed in Supplemental Table S1 on a StepOnePlus Real-Time PCR system (Life Technologies, Carlsbad, CA, USA). The standard curve method was used for quantification and expression levels were normalized against *GADPH*.

### Supplementary Information

Supplementary Information.

## Data Availability

CCIVR2 is available from github at https://github.com/CCIVR/ccivr2. The scripts that support the analysis, including "autoccivr2.py", "count_cg_lr.py", and "count_cg_pg.py", are located at https://github.com/CCIVR/suzuki2023_ccivr2.
