# Supplementary material for: CCIVR2 facilitates comprehensive identification of both overlapping and non-overlapping antisense transcripts within specified regions
Source: Sci Rep. 2023 Sep 8;13:14807. doi: 10.1038/s41598-023-42044-x (PMC10491648; doi:10.1038/s41598-023-42044-x)
Supplement: Supplementary file 1 — Supplementary Information. [file 41598_2023_42044_MOESM1_ESM.pdf]

Supplementary information

CCIVR2 facilitates comprehensive identification of both overlapping and non-overlapping antisense transcripts within specified regions

Maya Suzuki[1,4], Satoshi Sakai[1], Kosuke Ota[1], Yuki Bando[2], Chiharu Uchida[3], Hiroyuki Niida[1], Masatoshi Kitagawa[1], Tatsuya Ohhata[1,4,*]

[1]Department of Molecular Biology, Hamamatsu University School of Medicine, Hamamatsu, Shizuoka 431-3192, Japan

[2]Department of Organ and Tissue Anatomy, Hamamatsu University School of Medicine, Hamamatsu, Shizuoka 431-3192, Japan

[3]Advanced Research Facilities & Services, Preeminent Medical Photonics Education & Research Center, Hamamatsu University School of Medicine, Hamamatsu, Shizuoka 431-3192, Japan

[4]These authors contributed equally

*Correspondence: ohhata@hama-med.ac.jp (T.O.)

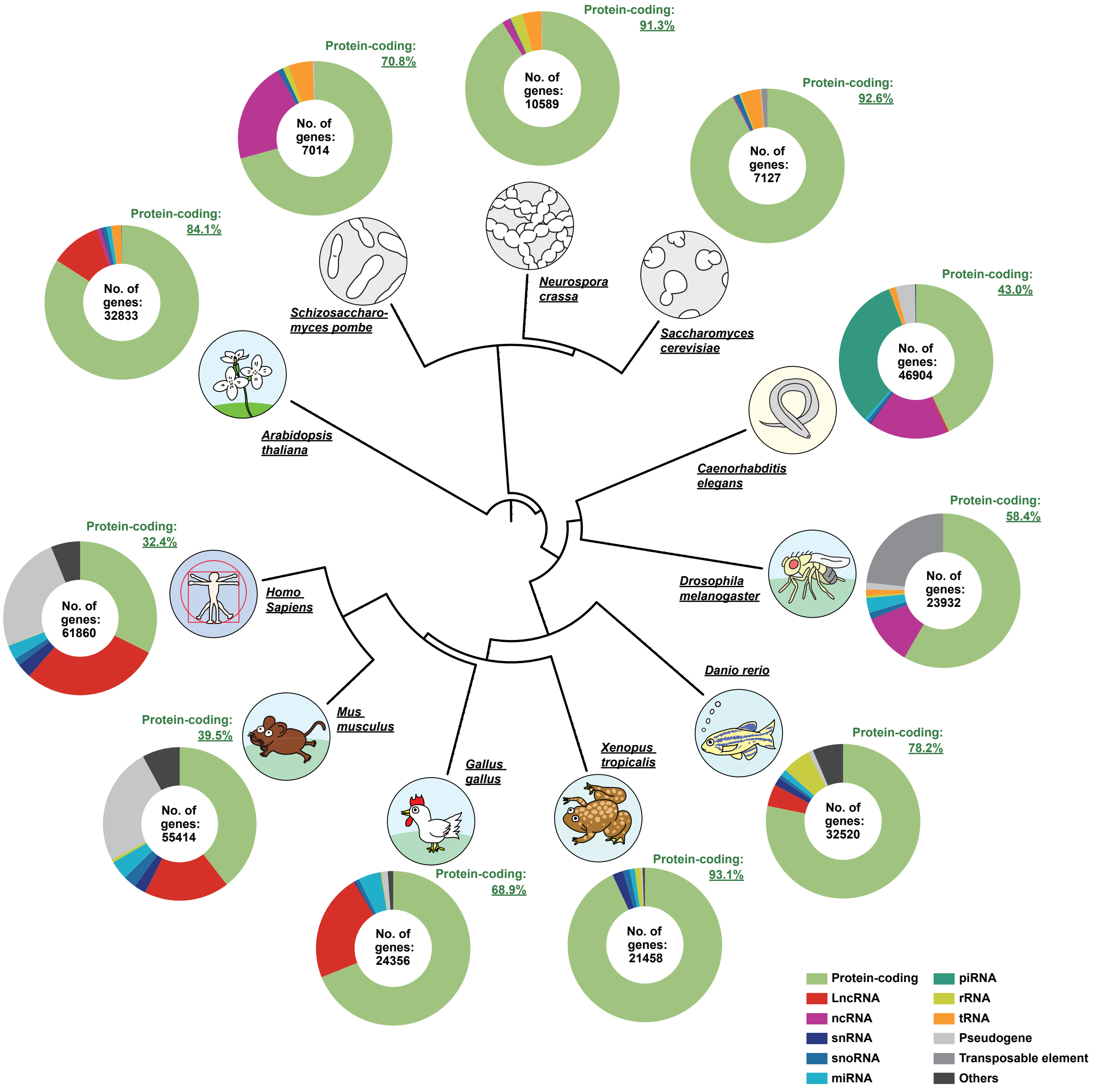

Figure S1 Types of RNA in representative model organisms

The types of RNA, as well as the percentage of protein coding genes in eleven different model organisms, are shown. The Ensembl/Ensembl_plant/Ensembl_fungi genome version used is mentioned in Figure 2. The phylogenetic tree was generated by phyloT_v2 (https://phylot.biobyte.de).

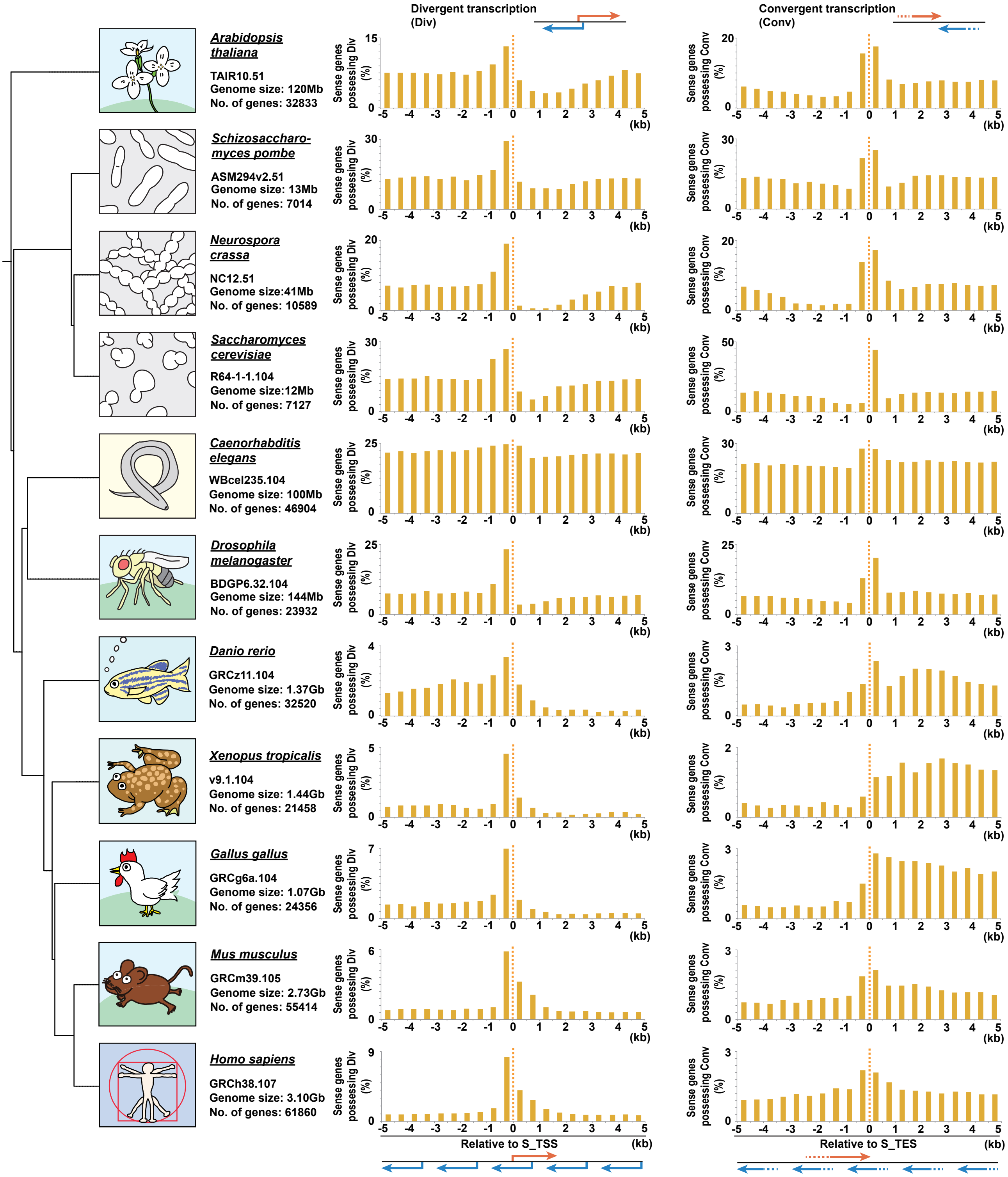

Figure S2. Identification of divergent and convergent transcription within the specified region: all annotated gene types from representative model organisms

The percentage of sense transcripts that possess a TSS or TES of an antisense transcript in the specified region relative to the TSS or TES of the sense transcripts is shown. The specified regions are each 500 bp. The results of all annotated gene types from 11 different model organisms are shown. Red and blue arrows represent sense transcripts and their antisense transcripts, respectively. The Ensembl/Ensembl_plant/Ensembl_fungi genome version used is shown below the name of each species. The phylogenetic tree was generated using phyloT_v2 (https://phylot.biobyte.de).

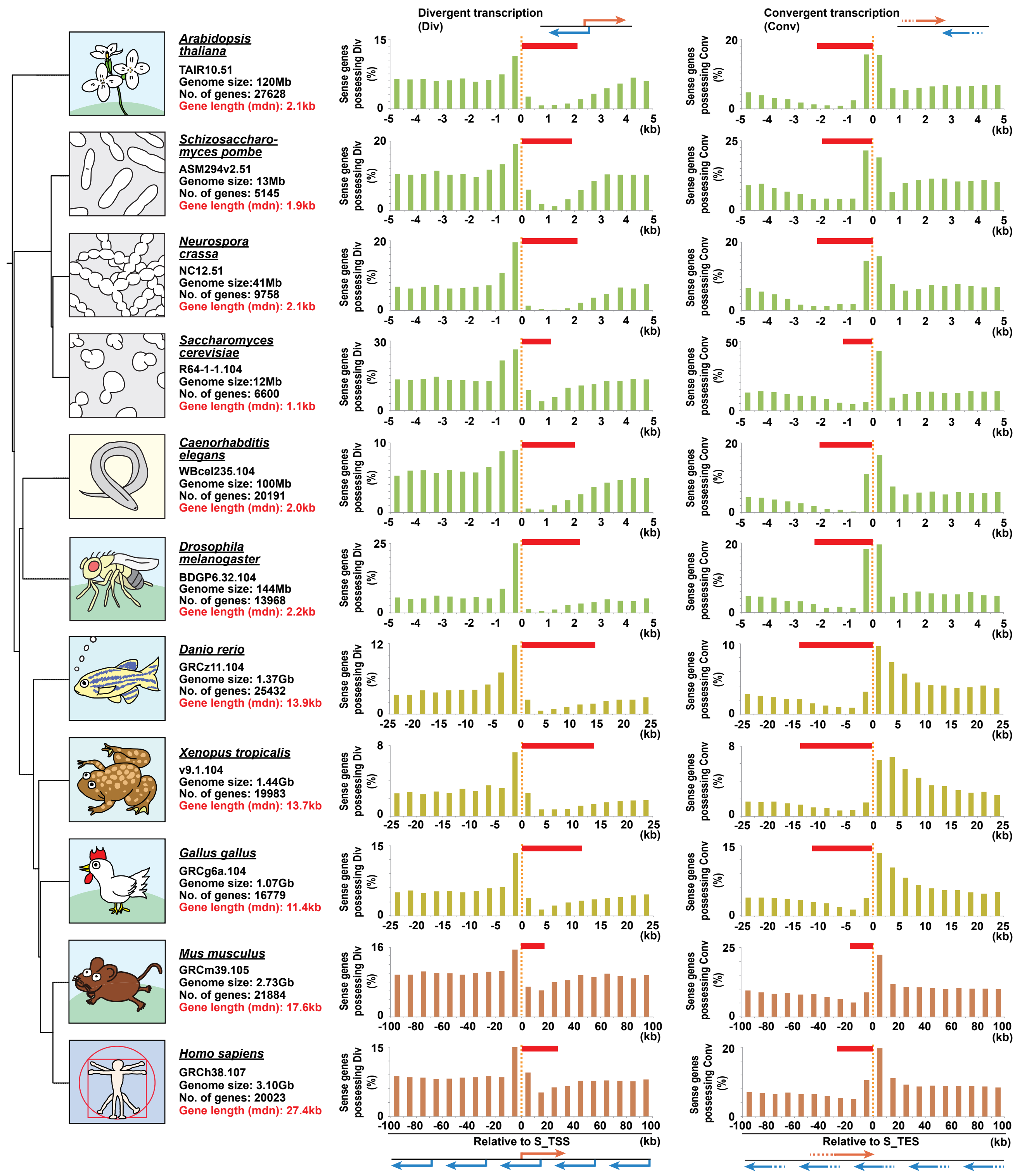

Figure S3. Identification of divergent and convergent transcription within the specified region with respect to gene length in representative model organisms

The percentage of sense transcripts that possess a TSS or TES of an antisense transcript in the specified region relative to the TSS or TES of the sense transcripts is shown. The range of analysis varies from species to species, reflecting the different gene lengths in each species. The horizontal red bars represent the median (mdn) gene length for each species. The phylogenetic tree was generated using phyloT_v2 (https://phylot.biobyte.de).

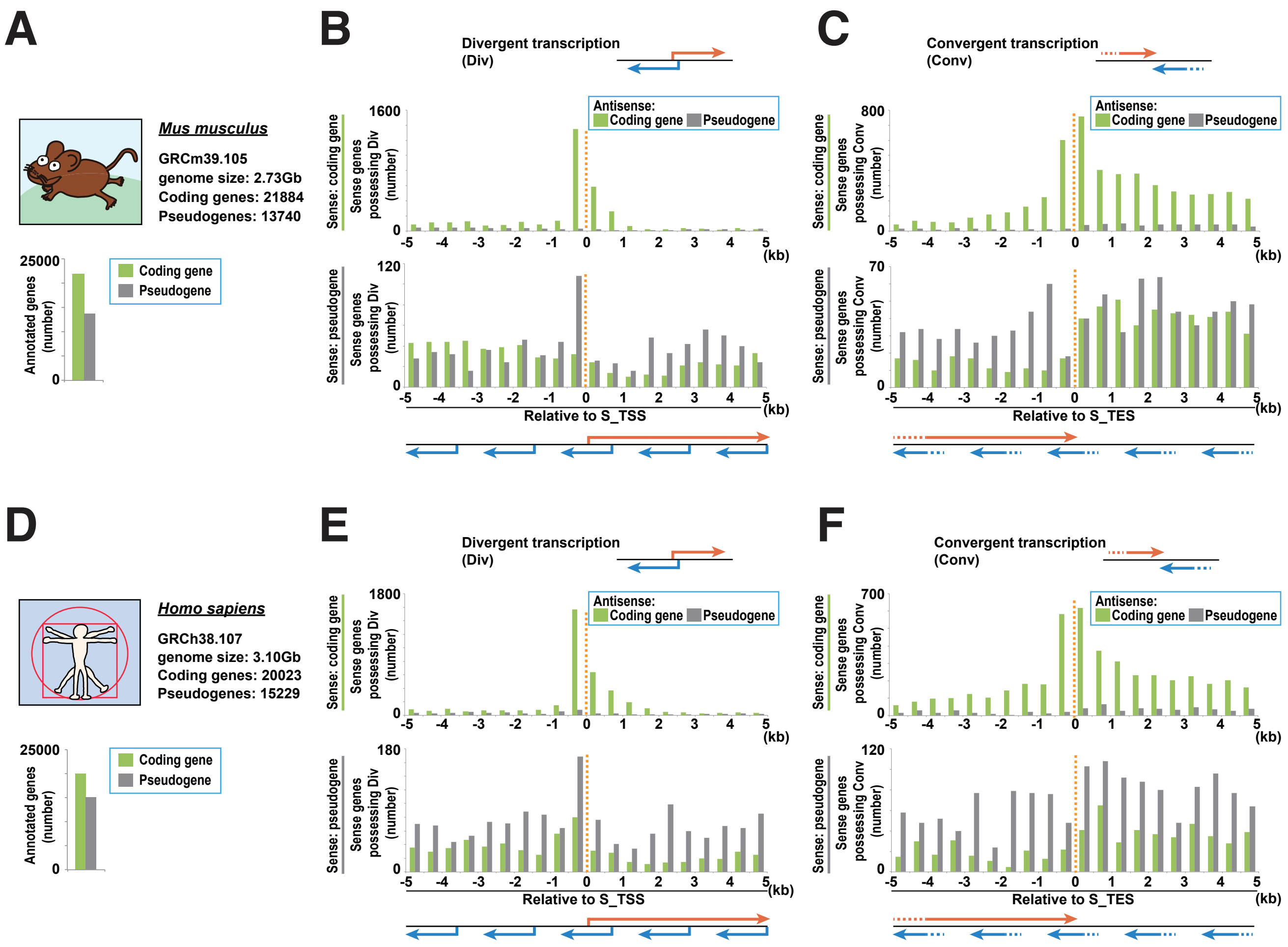

Figure S4. Comparison of divergent and convergent transcription between coding genes and pseudogenes in mouse and human

A-C Identification of divergent and convergent transcription with protein-coding genes and pseudogenes in mice. The Ensembl genome version used in this study, the genome size, and the number of coding genes and pseudogenes annotated in the database are shown below the name of the species (A), and the number of sense transcripts that possess a TSS (B) or TES (C) of an antisense transcript in the specified region relative to the TSS (B) or TES (C) of the sense transcripts is shown. Their gene biotypes (*i.e.* coding gene or pseudogene) are specified in each panel. The width of each specified region is 500 bp. Red and blue arrows represent sense transcripts and their antisense transcripts, respectively.

D-F Identification of divergent and convergent transcription with protein-coding genes and pseudogenes in human, as shown in (A–C).

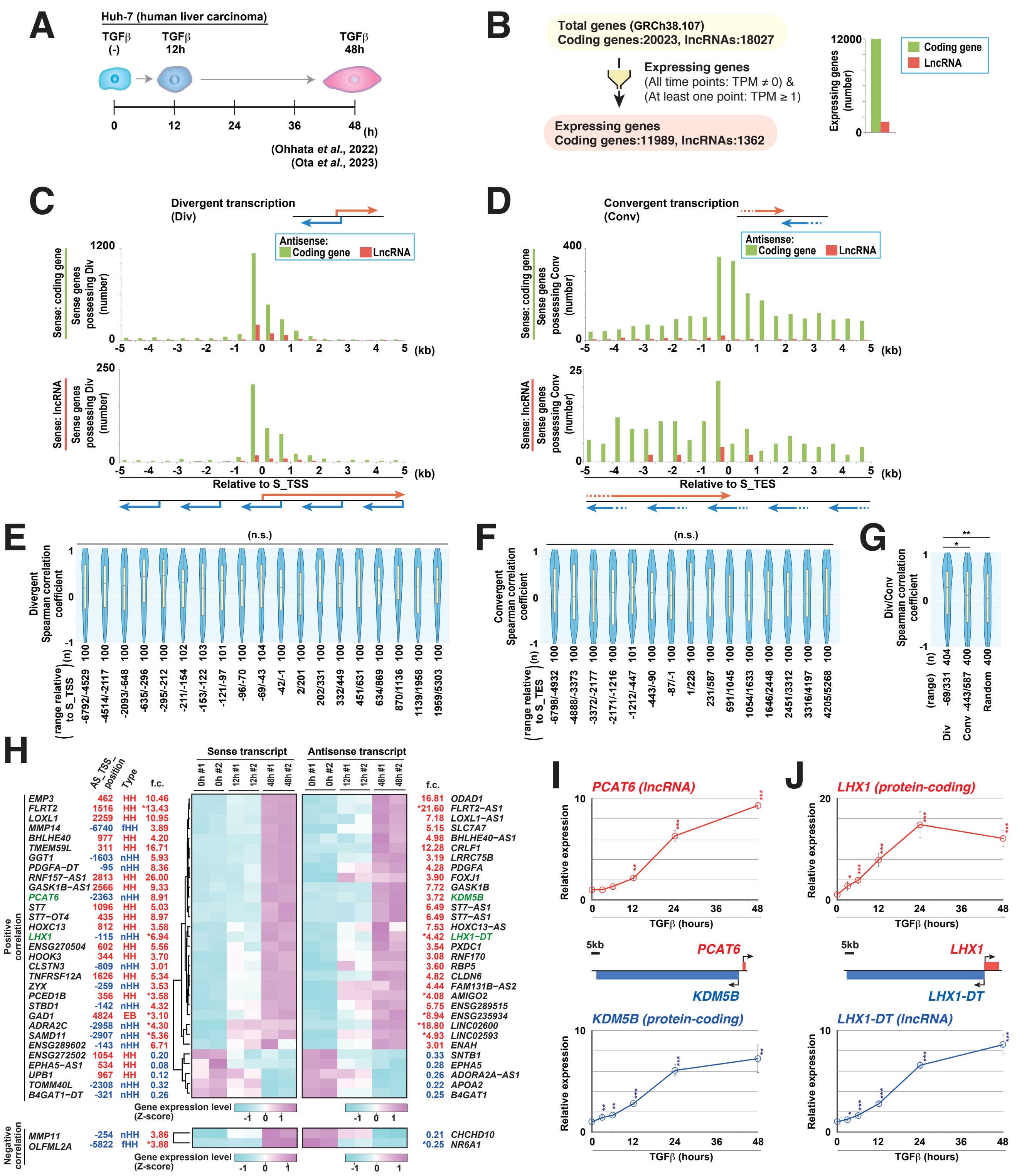

Figure S5. Example of CCIVR2 analysis II: identification of divergent transcription with TGFβ stimulation

A Schematic representation of TGFβ stimulation of Huh-7 cells[1,2].

B Selection of expressing genes with TGFβ stimulation. The definition and number of expressing genes are shown.

C,D Identification of divergent (C) and convergent (D) transcription from expressing genes with TGFβ stimulation, as shown in Figure 3.

E-G Spearman correlation coefficient analysis, comparing the gene expression dynamics between sense and antisense transcripts with TGFβ stimulation. Box-and-whisker plots are shown within the violin plots for the specified regions of divergent (E), convergent (F), and divergent/convergent (G) transcription. n.s.: not significant, **p < 0.01, Steel-Dwass's multiple comparison test.

H Heatmap analysis of senes/antisense transcript pairs from divergent transcription. Up and down genes were selected when the fold change (f.c.) in expression was more than 3.00 and less than 0.33 compared with no TGFβ stimulation, respectively, with statistical significance (padj) < 0.05, at the time points of 12 h or 48 h. Fold change (f.c.) with an asterisk (*) indicates differential expression after TGFβ stimulation for 12 h and the other values are for 48 h after TGFβ stimulation. The distance between sense and antisense transcript TSSs is shown (AS_TSS_position). The genes in green font were chosen for further studies.

I,J Expression dynamics of *PCAT6*/*KDM5B* (I) and *LHX1*/*LHX1-DT* (J) sense/antisense transcript pairs with TGFβ stimulation for 48 h, confirmed by RT-qPCR. No TGFβ stimulation (0 h) is set at 1 and means ± SD normalized to *GAPDH* across biological replicates (n = 3) are shown. *p < 0.05, **p < 0.01, ***p < 0.001, compared with or without TGFβ stimulation (0 h); Student's t-test.< 0.001, compared to each of without TGFβ stimulation (0 h); Student's t-test.

Supplemental Table S1. Information for primer sequences

PCR products	Primer sequence	Intron	Expected length	Application	References
PCAT6		No	70bp	RT-qPCR (Fig. S5I)	
h_PCAT6_1_(+)_18	AAACCGCCCTCATTTGTG				
h_PCAT6_1_(-)_20	GACCGAATGAGGATGGAGAC				
KDM5B		No	132bp	RT-qPCR (Fig. S5I)	
h_KDM5B_1_(+)_20	AATCCAGCTCCCTGTTTGTG				
h_KDM5B_1_(-)_20	TGACCTCACCATTCCATTCC				
LHX1		No	94bp	RT-qPCR (Fig. S5J)	
h_LHX1_1_(+)_21	AGCCCAGAATCCAGAGAGAAG				
h_LHX1_1_(-)_20	GGCATCAGGCATTTCATAGG				
LHX1-DT		No	77bp	RT-qPCR (Fig. S5J)	
h_LHX1-DT_1_(+)_20	GCATGACTTTGAAGGGAAGG				
h_LHX1-DT_1_(-)_20	TTCCAGATGAGTGGGGAAAC				
GAPDH		No	87bp	RT-qPCR (Fig. S5I, S5J)	3
GAPDH-22-F	TGCACCACCAACTGCTTAGC				
GAPDH-22-R	GGCATGGACTGTGGTCATGAG				

References

1. Ohhata, T. *et al.* CCIVR facilitates comprehensive identification of cis-natural antisense transcripts with their structural characteristics and expression profiles. *Sci Rep* **12,** 15525 (2022).

2. Ota, K. *et al.* APOBEC3B expression is promoted by *lincNMR* collaborating with TGF-β-Smad pathway. *Carcinogenesis* **44,** 1–14 (2023).

3. Sakai, S. *et al.* Long noncoding RNA *ELIT-1* acts as a Smad3 cofactor to facilitate TGFβ/Smad signaling and promote epithelial-mesenchymal transition. *Cancer Res.* **79,** 2821–2838 (2019).
